# Human Metapneumovirus and Community-Acquired Respiratory Illness in Children

**DOI:** 10.3201/eid0905.020615

**Published:** 2003-05

**Authors:** Diego Vicente, Gustavo Cilla, Milagros Montes, Emilio Pérez-Trallero

**Affiliations:** * Hospital Donostia, San Sebastián, Spain; † Universidad del País Vasco, San Sebastián, Spain

**To the Editor:** Stockton et al. have reported the detection of human metapneumovirus (HMPV) by using reverse transcriptase-polymerase chain reaction (PCR) in patients with influenzalike illness (*1*). These authors examined specimens submitted from patients, mainly adults, during winter 2000–01 and identified HMPV in 2.2% of patients with influenzalike illness who had tested negative for influenza virus and human respiratory syncytial virus (HRSV). Although several papers have been published on HMPV infection in children (*2*–*4*), the real impact of this virus on the health of the pediatric population remains to be determined. The data we obtained in the present study support the epidemiologic findings of J. Stockton et al. (*1*) and reinforce the notion that HMPV is a human pathogen associated with community-acquired acute respiratory tract infection (ARTI).

We investigated the occurrence of HMPV in children <3 years of age with ARTI during two consecutive winter seasons (November 2000–February 2001 and November 2001–February 2002) as part of a study to detect respiratory viruses (HRSV, influenza A and B viruses, parainfluenza virus types 1–4, and adenovirus) among the pediatric population. The study population comprised 565 children who were brought to Hospital Donostia, San Sebastián, Spain, with reported symptoms of ARTI, most of which (>80%) affected the lower respiratory tract. Of these children, 379 were hospitalized and 186 were discharged without admission. Hospital Donostia belongs to the public health system and is the main referral hospital for a population of 9,500 children <3 years of age. More than 97% of hospitalizations of children in our region occur in this hospital.

Nasopharyngeal aspirates were obtained and processed for cell culture by using rapid shell vial techniques on the MDCK, A-549, and LLC-MK2 cell lines. RNA was then extracted from the original samples by using phenol-chloroform (TRIzol LS Reagent, Invitrogen Corp., Carlsbad, U.K.) and was converted into cDNA with random primers by using M-MuLV reverse transcriptase (USB Corp., Cleveland, OH). Nested PCR was performed to detect HRSV, influenza, and parainfluenza viruses as previously described (*5*,*6*). The remaining cDNA was frozen at –80°C until subsequent use. We tested for HMPV in all samples that tested negative for the previously studied viruses, as well as in 100 randomly selected study samples that were positive for one or more of these viruses. HMPV detection was performed by PCR by using 5 µL of stored cDNA with primers derived from the F gene under previously described conditions (*7*). The PCR product (450 bp) from the HMPV-positive samples was sequenced in an ABI PRISM 3100 Genetic Analyzer (Applied Biosystems, Foster City, CA).

In 411 (72.7%) of the 565 patients studied, at least one of the initially investigated viruses was detected. HRSV was found in 313 (55.4%) children, influenza in 44 (7.8%), parainfluenza in 36 (6.4%), and adenovirus in 32 (5.7%); 14 mixed infections were detected. Of 154 children with a negative result, HMPV detection was performed in 147 (95.5%), with a positive result in six children (4.1%). No HMPV was detected in any of the 100 samples previously positive for the initially studied respiratory viruses. Four of the six HMPV-positive children required hospitalization: a 7-month-old boy with pulmonary bronchodysplasia, rhinitis, and fever of 38.4°C (patient 1); a 20-month-old girl with previous obstructive pulmonary disease who had acute respiratory insufficiency along with generalized hypoventilation, crackles, wheezing, and radiologic images of air entrapment requiring bronchodilator administration (patient 2); a 16-month-old girl who had a febrile syndrome, basal crackles on pulmonary auscultation, and perihilar infiltrates (patient 3); and an 11-month-old boy with pneumonia of the upper left lobe (patient 4). The two remaining patients, a 7-month-old boy (patient 5) and a 9-month-old girl (patient 6), both with upper respiratory symptoms and clear chest, did not require hospitalization. In all six patients, outcome was favorable.

Analysis of the amplified sequences showed two clusters of HMPV. The first was composed of HMPV from patients 1, 3, 4, and 6 (GenBank accession nos. AY152846, AY152851, AY152850, and AY152847, respectively), and the second was composed of HMPV from patients 2 and 5 (GenBank accession nos. AY152849 and AY152848). The similarity among nucleotide sequences in the same cluster was >95% and oscillated from 86% to 88% when compared to those from a different cluster. During the second study season, we observed circulation of both clusters. When we compared these sequences of HMPV F gene obtained in Spain with those recently described in North America (*7*), we found that the sequences of the first cluster showed >95% similarity with the isolate CAN97-83 (GenBank accession no. AY145296), and the sequences from the second cluster showed >95% similarity with isolates CAN98-73 to CAN98-79 (GenBank accession nos. AY145287–AY145293), connecting the Canadian isolates to two well characterized groups of HMPV. Our results suggest that in Spain, as well as in other places in the world (*2*,*7*), two major HMPV groups exist. The severity of the episodes observed varied from mild upper respiratory symptoms to severe infections requiring hospitalization for 2–6 days. Overall, as reported by other authors (*2*,*8*), the clinical picture provoked by HMPV was indistinguishable from that of other respiratory viruses. The fact that HMPV was not detected in any of the samples from patients also positive for other respiratory viruses suggests that coinfection is infrequent. The data reported in our study, obtained during two consecutive winter seasons in a pediatric population of southern Europe, allow us to estimate that the incidence of moderate or severe respiratory infections caused by HMPV is low and that the impact of the other respiratory viruses is considerably greater. Despite these results, we think that this new respiratory pathogen warrants surveillance. HMPV appears to be capable of provoking severe infections, and its role in human respiratory infections is still poorly understood.
